# Conservation of Gene Cassettes among Diverse Viruses of the Human Gut

**DOI:** 10.1371/journal.pone.0042342

**Published:** 2012-08-10

**Authors:** Samuel Minot, Gary D. Wu, James D. Lewis, Frederic D. Bushman

**Affiliations:** 1 Department of Microbiology, Perelman School of Medicine at the University of Pennsylvania, Philadelphia, Pennsylvania, United States of America; 2 Division of Gastroenterology, Perelman School of Medicine at the University of Pennsylvania, Philadelphia, Pennsylvania, United States of America; 3 Center for Clinical Epidemiology and Biostatistics, Perelman School of Medicine at the University of Pennsylvania, Philadelphia, Pennsylvania, United States of America; Universidad Miguel Hernandez, Spain

## Abstract

Viruses are a crucial component of the human microbiome, but large population sizes, high sequence diversity, and high frequencies of novel genes have hindered genomic analysis by high-throughput sequencing. Here we investigate approaches to metagenomic assembly to probe genome structure in a sample of 5.6 Gb of gut viral DNA sequence from six individuals. Tests showed that a new pipeline based on DeBruijn graph assembly yielded longer contigs that were able to recruit more reads than the equivalent non-optimized, single-pass approach. To characterize gene content, the database of viral RefSeq proteins was compared to the assembled viral contigs, generating a bipartite graph with functional cassettes linking together viral contigs, which revealed a high degree of connectivity between diverse genomes involving multiple genes of the same functional class. In a second step, open reading frames were grouped by their co-occurrence on contigs in a database-independent manner, revealing conserved cassettes of co-oriented ORFs. These methods reveal that free-living bacteriophages, while usually dissimilar at the nucleotide level, often have significant similarity at the level of encoded amino acid motifs, gene order, and gene orientation. These findings thus connect contemporary metagenomic analysis with classical studies of bacteriophage genomic cassettes. Software is available at https://sourceforge.net/projects/optitdba/.

## Introduction

Advances in DNA sequencing technology have made it possible to characterize microbial communities using extremely large numbers of short sequence reads [1]–[3]. This offers a powerful tool for interrogating complex communities of uncultured organisms, but analyzing the shotgun sequence data from mixtures of organisms poses considerable computational challenges [4]–[7]. Here we address the problem of assembling genomes from complex viral communities to investigate conserved features of gene content and order.

Viral communities influence microbial populations and human health, but their study is hampered by a large degree of uncharacterized sequence diversity. It has been estimated that 0.0002% of the global viral gene pool has been sequenced [8] and deep sequencing of viruses purified from the environment typically yields a large majority of unidentified sequences [2], [3], [9]–[11]. Thus efficient studies of viral populations using sequence-based surveys depends on the efficient computational assembly of individual reads into large genome fragments without reference to known genomes.

The assembly of mixed viral reads presents a number of challenges. Viral genomes are small but range widely in size, from 5 kb to >1 Mb [12]–[14], so size cannot easily be used to assess genome completion. Different viral genomes can be present in widely differing proportions [15], [16], complicating the use of coverage to judge correct assembly. Viral genomes can also evolve quickly, including frequent recombinational exchange of protein-coding cassettes [17]–[19] and high rates of nucleotide substitution [11], further confusing assembly.

However, many viral genomes are either circular, such as φX174 [20], or are circularly permuted, such as T4, which includes 1.02 genome copies in each viral head [21]. Thus assembly of reads into circles indicates probable completion of the genome sequence of a circular virus. Circularity not been previously been used widely to improve viral sequence assembly, probably because most previous virome studies did not acquire enough sequence data to allow routine closure of circular assemblies.

**Figure 1:**
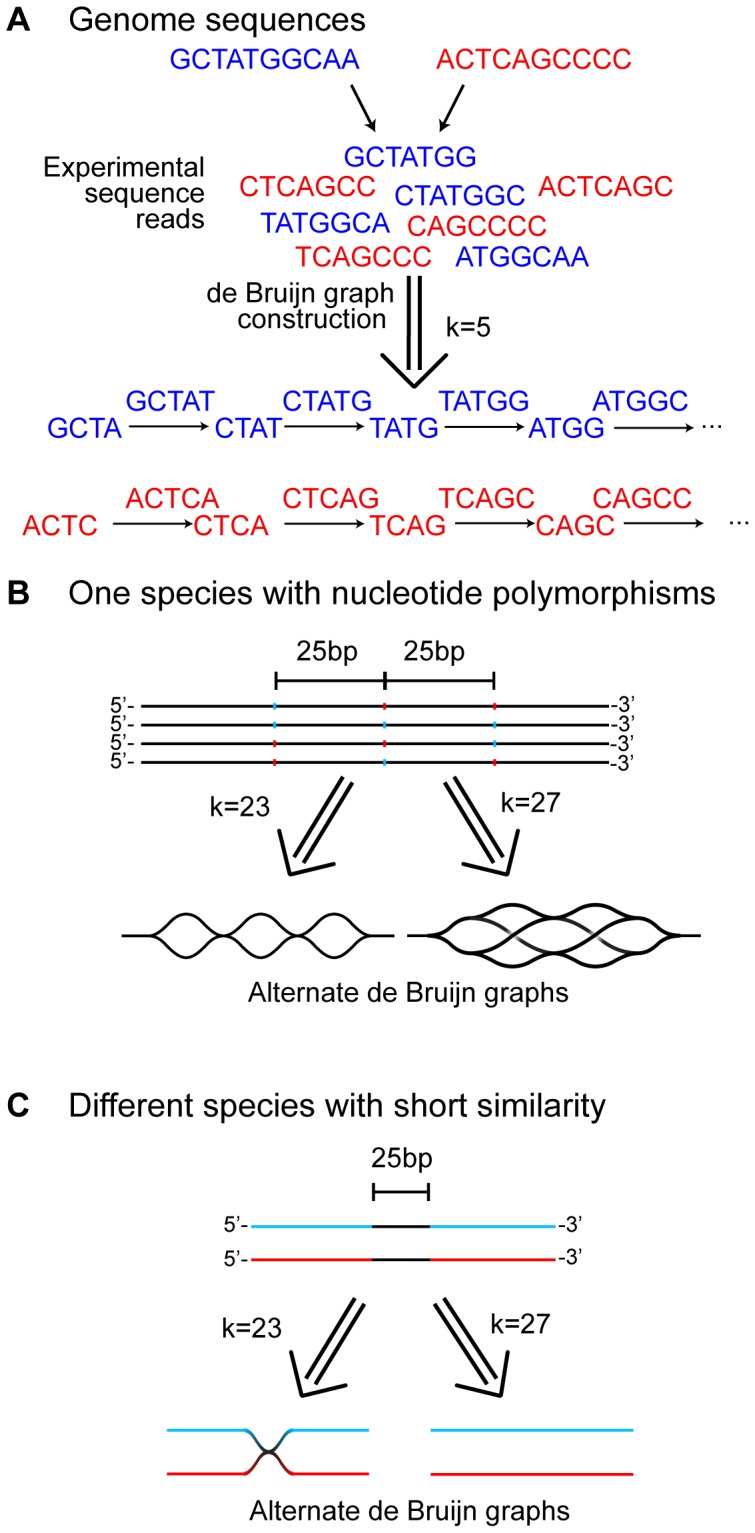
The de Bruijn graph assembly method and the influence of genomic variation on de Bruijn graph complexity. A) Shotgun sequences are produced from two different genomes (shown in blue and red at the top). Those sequences are used to construct a de Bruijn graph, where nodes are formed by all possible sequences of length k-1 (in this case 4 bases), which are connected by edges of length k (5 bases). Since there are no 4mers shared between these two example genomes, the resulting de Bruijn subgraphs are separate. B) Nucleotide polymorphisms are better resolved by short kmers. We consider a mixture of four genomes, each with three polymorphic positions separated by 25 bp. The identity at each polymorphic position is represented by either blue or red to indicate different nucleotides. At all other positions the genomes are identical. The de Bruijn graph that is constructed from this mixture of genomes using a kmer of 23 is shown on the left, where three independent bubbles form around each polymorphic position. The equivalent graph at k = 27 is shown on the right, where three independent sets of bubbles overlap, forming a more complex and suboptimal graph structure. C) Short regions of similarity are better resolved by long kmers. We consider a mixture of two genomes which are entirely different except for a 25 bp region of sequence identity (shown in black). The de Bruijn graph that is constructed from this mixture at k = 23 is shown on the left, where the two resulting subgraphs intersect at the 23mer of similarity. The de Bruijn graph at k = 27 is shown on the right, where the two resulting subgraphs (corresponding to the two genomes) do not intersect, since they have no 26mer in common. The examples in B and C together illustrate how different kmers can be optimal for assembling graphs with different types of polymorphisms.

The problem of *de novo* assembly of high-throughput sequencing datasets has been greatly aided by the development of de Bruijn graph assemblers [22]–[27]. In the de Bruijn graph method, extremely large sets of short sequences (such as those generated by Illumina HiSeq technology) can be assembled into complete and partial genomes by mapping them onto a de Bruijn graph (Fig 1A) [24], [28]. Each read is computationally fragmented into sequences of length k (the so-called ‘kmer’), then each kmer is used to form an edge between nodes corresponding to sequences of length k-1. By drawing such edges for every read in the dataset, one constructs a de Bruijn graph, which contains the information necessary to reconstruct the genome sequences that gave rise to the graph. In a subsequent step, a consensus contig sequence is constructed from the de Bruijn graph, which involves ‘popping’ (condensing) bubbles into linear segments, trimming branches, resolving repeats, and more complex operations to generate a linear graph [4]. Assembly by this method scales linearly with increasing sequence number, while the more familiar overlap method of assembly scales exponentially, explaining why the de Bruijn graph method is used for very large data sets [22].

However, complexities within the sequence population, such as nucleotide polymorphisms or short sequence repeats, can introduce misleading connections in the de Bruijn graph [5], [29]. In Figure 1 we demonstrate how the optimal kmer – i. e. one that minimizes misleading connections – depends on the nature of the underlying sequence. A set of genomes with three independent SNPs separated by 25 bp (Figure 1B) will produce a de Bruijn graph with three isolated bubbles at a kmer of 23, while it will produce a much more complex structure at a kmer of 27. In contrast, two unrelated genomes with 25 bp of identical sequence (Figure 1C) will be joined together at a kmer of 23 bp, but not at one of 27 bp. These examples demonstrate how the difficulty of parsing a de Bruijn graph depends both on the nature of the underlying polymorphism and the kmer value used.

Thus in a mixture of multiple microbial genomes, it is likely that the optimal kmer value for assembly will vary [5], [30]. One group [5] found that combining the assemblies constructed across a range of kmer values yielded a large number of long contigs, but that these contigs did not faithfully represent the underlying genomes. Another group developed IDBA, which performs sequential assemblies while stepping through kmers of increasing lengths [30]. At each kmer value, IDBA removes the best contigs and the reads used to make those contigs. A metagenomic version of this program, MetaIDBA, has been developed [29].

In this paper our goal is to find patterns of genome conservation in the highly diverse collection of viruses found in the human gut [2], [3], [11], [31], [32]. We implement an optimized iterative de Bruijn graph assembly approach, significantly increasing the length and depth of the assembled contigs compared with previous virome studies. We present results for 5.6 Gb of Illumina paired-end sequence data from six human gut virome samples (a subset of samples reported initially in [11]). While only a minority of the assembled ORFs in the sample could be annotated – emphasizing the vast diversity of gut viral populations – the annotated ORFs tended to group by predicted function. Moreover, many ORFs could be clustered into inferred cassettes with conserved gene order and orientation. Thus our analysis emphasizes the extreme variation in gut bacteriophage populations across individuals, and that viral genomes are organized in conserved multi-gene cassettes.

## Results

### Assembly of viral contigs

**Figure 2:**
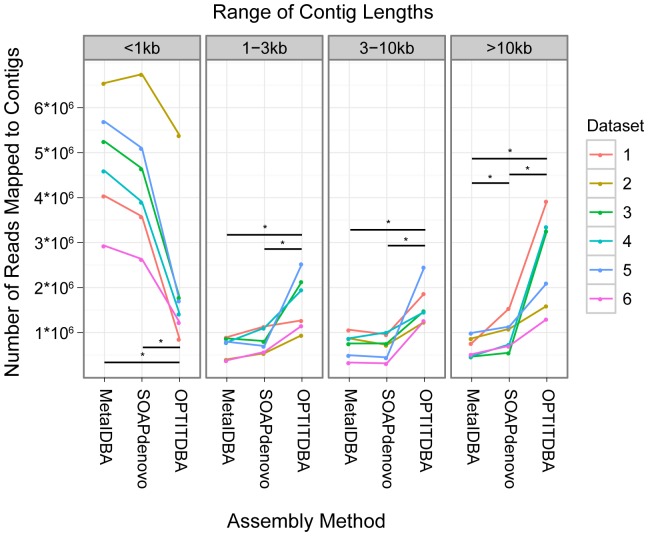
Comparison of assembly methods by read alignment. The vertical axis indicates the number of reads from each dataset that align to contigs of different size classes (either less than 1 kb, between 1 kb and 3 kb, between 3 and 10 kb, or longer than 10 kb). The horizontal axis separates assembly method. Each dataset is indicated by color (see key on right; numbers indicate gut virome communities from different human subjects). * indicates p<0.05 by Wilcoxon signed-rank test for the indicated pair of assembly methods.

In order to analyze protein conservation among viruses derived from mixed environmental samples, it is necessary to generate contigs that most closely approximate complete viral genomes. To generate long contigs that faithfully represent the underlying genome structure, we developed and employed an optimized iterative de Bruijn graph assembly approach (OPTITDBA). We compared this assembly method to two previously published methods (SOAPdenovo and MetaIDBA) using 5.6 Gb of Illumina HiSeq data (100 bp paired end reads) derived from stool virome samples from six healthy human subjects [11]. An example of assembly for samples from one of the six subjects is shown in Fig. S1. We found that assembly using our iterative method (OPTITDBA) resulted in fewer reads mapped to contigs less than 1 kb in length, and more reads mapped to contigs in each of the three longer size classes (1–3 kb, 3–10 kb, and >10 kb) (Fig 2), performing better than either SOAPdenovo or MetaIDBA (p<0.05; Wilcox signed-rank test).

In order to measure the accuracy of this method, we used synthetic viral communities composed from previously sequenced virome samples. Of the 6 subjects in this dataset, one contained sequences that align closely to Human Papilloma Virus type 6b (as reported previously in [11]). These reads were added in varying amounts to a collection of reads from a subject completely lacking HPV reads, and the resulting synthetic datasets were used to assess quality of assembly. We assembled these synthetic viral communities using OPTITDBA, MetaIDBA, or SOAPdenovo and compared the efficiency of HPV recovery (measured in this case as the length of the longest HPV contig as a proportion of the whole HPV genome). For every level of sequencing (6, 13, 19, and 23X coverage), the HPV genome was better assembled using this pipeline than using the single pass SOAPdenovo assembly (p<0.0005; Wilcox signed-rank test) (Fig. S2). On average, this pipeline performed 61% better than the corresponding single assembly using SOAPdenovo. There was no significant difference in HPV genome recovery between MetaIDBA and OPTITDBA, though our pipeline was better than MetaIDBA in producing contigs that recruited the maximum number of reads. In summary, OPTITDBA assembled viral reads into longer contigs at no cost to accuracy in the reconstruction of the control genome.

### Network analysis of bacteriophage proteins

In order to characterize the assembled viral genomes, we predicted open reading frames (ORFs) using Glimmer, yielding 29,017 ORFs longer than 100 bp from the 6 datasets. Of these, only 3,066 had similarity at a cutoff of E<10^−10^ to the RefSeq collection of viral proteins (10.6%). At a more stringent cutoff of E<10^−50^, only 690 ORFs were similar (2.4%). Searching for conserved amino acid motifs contained within the Conserved Domain Databases (CDD) yielded 3,374 ORFs with a match in the CDD at E<10^−10^ (11.6%), but only 777 with a match at E<10^−50^(2.7%).

**Figure 3:**
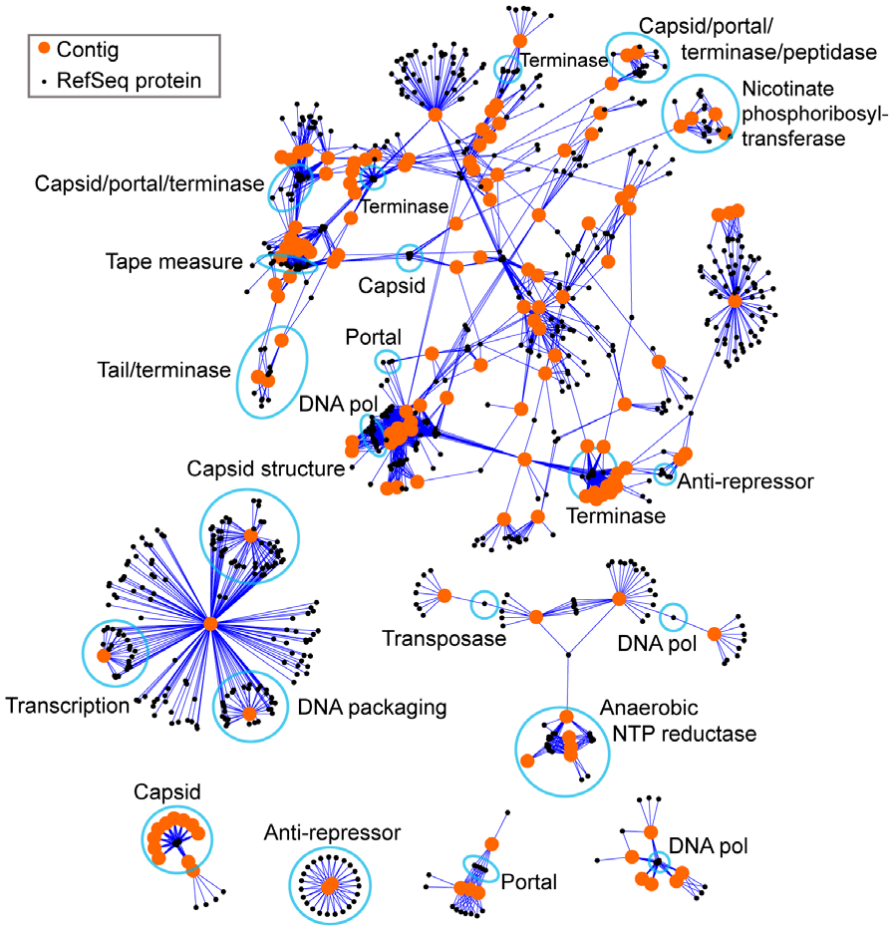
Network based annotation of viral contigs. Orange circles represent viral contigs no shorter than 3 kb. Black circles represent proteins in the RefSeq viral database. RefSeq proteins are connected to viral contigs when an ORF encoded by that contig resembles that protein at E<10^−50^ (blastp). Blue outlines indicate groups of RefSeq proteins and ORFs from contigs that share the function indicated by the adjacent label.

In order to investigate which of these RefSeq annotations were shared among contig-encoded ORFs, we carried out a network analysis (Fig. 3). The nodes in this network represent either contigs (orange circles) or RefSeq viral proteins (smaller black circles). Edges (connections) are drawn between contigs and RefSeq proteins when an ORF (encoded by a contig) is highly similar to a RefSeq protein (E<10^−50^). Groups of RefSeq proteins that are similar to multiple contigs are highlighted by light blue ovals. While in some cases these groups of reference proteins encode only a single function (in which case they are likely all similar to a single ORF on each of the indicated contigs), in others there are multiple predicted functions (in which case there is a similar collection of genes found on all of the indicated contigs). For example, multiple contigs are linked by genes encoding both capsid and terminase proteins, while others are linked by genes encoding transcription and DNA packaging functions. These examples parallel classical studies which showed that bacteriophage genomes are often organized into cassettes of functionally related genes [33]–[35].

### Bacteriophage genomes contain conserved cassettes encoded by divergent nucleic acid sequences

After finding only a low frequency of similarities between ORFs in viral contigs and database sequences, we searched for conserved gene cassettes in a database-independent manner. ORFs were compared within the assembled sequences to find encoded amino acids sequences that were repeated among multiple contigs, which we refer to as protein-coding families. Of the 29,007 predicted ORFs, 16,944 (58%) were found to be members of families, that is, ORFs on different contigs showed alignments with at least 30% identity. The degree to which proteins cluster into families strongly depends upon the alignment cutoff that is used. The limit of 30% was chosen to include dissimilar but related groups of proteins, close to the limit of detection of homology. Identifying specific organisms in metagenomic data would typically use a much higher threshold to yield high confidence assignments. A total of 2,961 families contained 2 ORFs each. The largest family contained 25 ORFs. Of these 5,135 protein-coding families, only 1,287 (25%) had any similarity to the Conserved Domain Database, emphasizing the amount of unexplored diversity in genes of the gut virome.

Relationships among these protein-encoding families were interrogated by grouping families into cassettes, consisting of different families that were found on the same group of contigs. We found 28 types of cassettes that contained from 2 to 8 protein-coding families. On average, the amount of each contig that was covered by each cassette was 1.6 kb, ranging from 105 bp to 11.5 kb. The mean proportion of each contig that was covered by a cassette was 27%, ranging from 1% to 90%. The most common cassette was found on 20 contigs generated from all six subjects studied. Of the 16,944 ORFs found in families, 651 (4%) were found in cassettes (Table 1). While a small proportion of the total number of predicted ORFs were grouped into cassettes, this accounted for a disproportionately large amount of the input sequence reads. The contigs containing at least one ORF accounted for 3.1*10^7^ reads. The contigs containing a cassette accounted for 5.9*10^6^ reads, or 18% of all contigs (Table 2). Therefore while the proportion of contigs that harbor cassettes is relatively small, contigs with cassettes represent highly abundant lineages.

**Table 1:** ORFs in families and cassettes.

	Dataset						
	1	2	3	4	5	6	Total
Contigs	8403	13258	4755	6067	4375	2415	39273
ORFs	9507	4508	3143	5618	4009	2232	29007
ORFs in families	5980	3056	2139	3648	2825	1527	16944
ORFs in cassettes	118	135	106	116	107	74	651

The number and proportion of ORFs predicted in each dataset that belong to protein-coding families (i.e. are not unique), and/or belong to cassettes (groups of protein-coding families that are found on the same set of contigs.

**Table 2:** Contigs and reads that form cassettes.

Contig Criteria	Contigs	Reads that align to those contigs
With at least 1 ORF	10032	31883951
With at least 1 ORF family	7024	29697888
With at least 1 cassette	326	5886117

The number of contigs, and the number of reads that align to those contigs, that contain at least 1 ORF, more than 1 ORF, at least 1 ORF family, and/or at least 1 cassette. The percentage of the total number of reads that align to contigs with at least 1 ORF is shown in parentheses.

Bacteriophage cassettes commonly show conserved gene orientation as well as conserved gene type, so we investigated orientation as well. The degree of co-orientation among protein-coding regions in cassettes was found to be high, with an average co-orientation score of 99% (compared to 25% co-orientation expected by chance), providing strong support for cassette structure.

**Figure 4:**
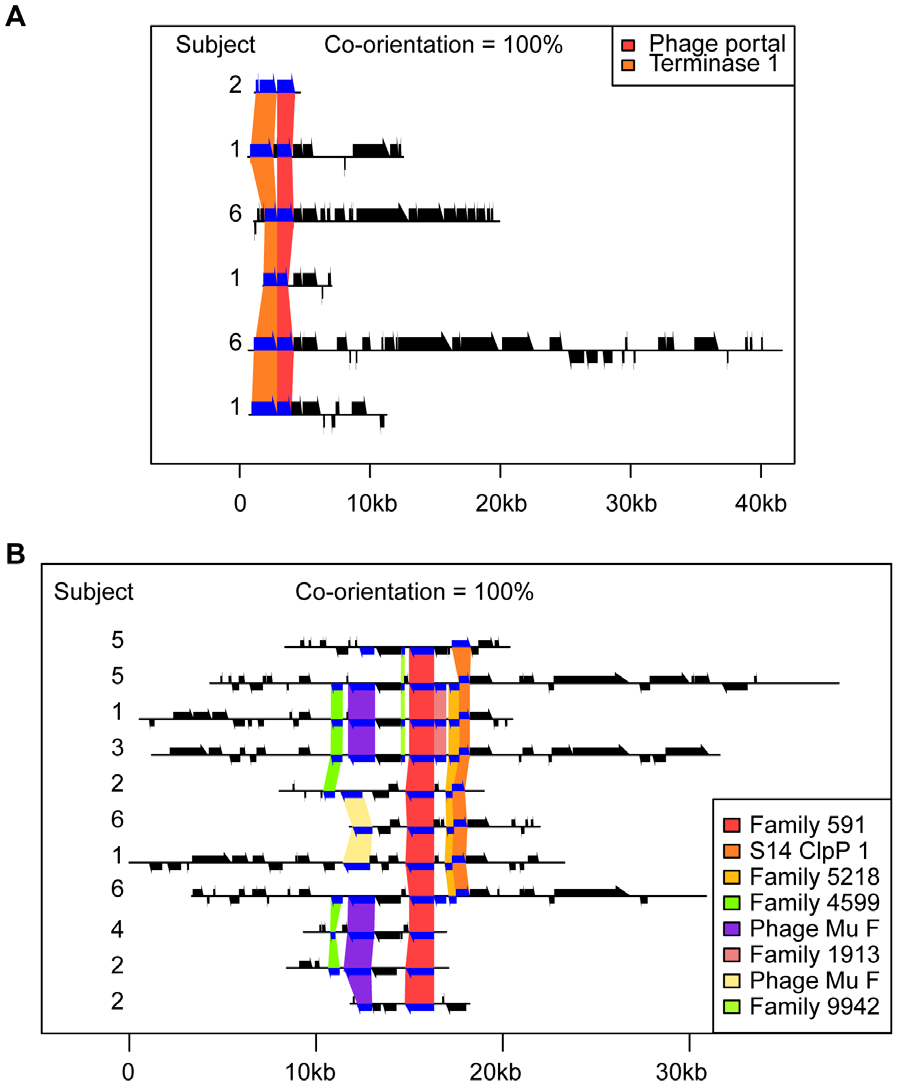
Two examples of phage cassettes. Contigs are shown as horizontal black lines, ORFs on those contigs are shown by black arrows above and below those lines, and the organization of those ORFs into protein-coding families is shown with colored boxes. The subject that each contig was assembled from is shown on the left of each panel. When a protein-coding family was functionally annotated according to its similarity with the CDD, that annotation is listed in the legend. Otherwise a unique identification number is shown (e. g. Family 591). The co-orientation score describes the proportion of gene pairs that, when occurring together on multiple contigs, do so in the same relative orientation.

In a few cases, the proteins encoded within a cassette showed potentially related annotations, such as N-6 DNA methylase and DEAD-like helicase (Fig S3) or phage portal and terminase (Fig. 4A). In many cases, specific unannotated ORFs were repeatedly found near ORFs annotated as phage proteins. In one case, proteins with less than 30% amino acid identity between them (resulting in their being grouped in different families) were assigned the same functional annotation (Phage Mu F: morphogenesis-related protein) and located in the same functional cassette (Fig. 4B), suggesting preservation of protein function and genetic organization despite nucleotide and amino acid divergence (Fig. 4B).

## Discussion

One difficulty of studying viruses in the environment is that high-throughput sequencing data is difficult to interpret when high proportions of reads are unknown or unrecognizable. One way to address this problem is through *de novo* assembly, generating complete and partial genome sequences. For environmental bacteriophage this is usually necessary because previously sequenced and closely related genomes are generally not available. We demonstrated that optimizing the assembly process according to the characteristics of viral genomes improves the degree of assembly at no cost to accuracy. Using our assemblies, we found that the viral open reading frames often cluster in related cassettes, but that the cassettes show considerable sequence divergence among genomes.

In our assembly pipeline we have improved on iterative kmer based de Bruijn graph assembly for use with viral samples in three ways. 1) We picked the optimal kmer value to use at each iteration, rather than cycling once through kmers of increasing length. 2) We removed at each iteration the set of reads that aligned to the best contigs, not those reads that were used to construct those contigs, because due to the nature of the de Bruijn graph assembly process, the set of reads used to construct a contig may not fully contain the set of reads that align well to that contig. 3) We reasoned that circular sequences would represent complete viral genomes, either as circular genomes or circularly permuted genomes, and so used this also as a criterion for calling finished contigs.

We found in our analysis that the selected kmer value had a large influence on the resulting assembly, likely due to complexities in the underlying sequences (as shown in Fig. 1). For using a de Bruijn graph method to assemble single genomes, this emphasizes the importance of comparing multiple kmer sizes. The iterative method that we describe tests a range of all possible kmer values and retains the contigs that are best assembled at each step.

As a measure of the quality of assembly, we monitored correct assembly of Human Papillomavirus Type 6b (HPV), the one known virus in our data set. We found that our pipeline was better able to assemble a single contig matching HPV across a range of sequencing depths than was SOAPdenovo (the underlying assembly algorithm used in our pipeline) by ∼61%. Both our pipeline and MetaIDBA reconstructed HPV about equally well (however, as described below, our method yielded contigs explaining a larger proportion of the reads). The ability to reconstruct viral genomes present at low abundance is particularly important when trying to detect pathogens in sequence mixtures, such as in efforts to identify novel pathogens in samples from outbreaks of infectious diseases.

A more complicated challenge is assessing the quality of assembly of unknown viral genomes. One common metric for assessing assembly quality is the length of contigs produced (N50). However, a recent study [5] found that for one implementation of De Bruijn graph assembly of short sequences from known genomes, the method that yielded the highest N50 yielded the lowest similarity to the known genomes. Therefore we chose to measure how well the contigs explain the input data by mapping reads back to contigs, and counting the number of reads that mapped to contigs of different size classes, thereby generating an estimate of how well the assembly process reconstructed the primary data. We found that our pipeline performed better than MetaIDBA or SOAPdenovo.

Analysis of protein conservation emphasized the cassette structure of the viral genomes in our samples. We annotate the viral contigs by aligning new ORFs to available databases, and by identifying ORFs of unknown function that aligned with other ORFs in our data set. We found that viral ORF families often clustered in cassettes, where genes with similar sequences were almost always in the same orientations. Cassette structure has been well documented in many bacteriophage families [13], [33]–[37]–here we show that these structural patterns are accessible after assembly of metagenomic data.

A conjecture to explain the observed phage genome structure invokes pressure for sequence diversification from the CRISPR system. Many bacterial genomes harbor a series of repeated sequences spaced by short sequences derived from phage or plasmids, called CRISPR arrays [38]. The CRISPR arrays are transcribed, then the spacer sequence RNAs are used as recognition elements to program degradation of incoming sequence-complimentary DNA. Thus bacteriophages that infect CRISPR-containing hosts are regularly under pressure to alter their DNA sequences to evade attack. Assisting this, bacteriophage replication cycles can be as short as 20 minutes and burst sizes large, allowing rapid evolution. There are also a variety of other mechanisms that bacteria use to resist bacteriophage infection and may also promote viral escape through mutation. Constraining the allowable DNA substitutions, of course, is the requirement for proper function of the encoded proteins. In a few cases three dimensional structures have been determined for multiple phage proteins encoded in syntenic regions from functionally interchangeable cassettes, and the structures can be surprisingly similar given the low DNA and protein similarity. For example, the repressor and Cro proteins of Lambda, 434, and P22 show little similarity at the nucleic acid level (median identity 34%) or amino acid level (median identity 17%) [39], but share common alpha-helical structures and helix-turn-helix motifs [40], [41]. Thus the modules emerging from the metagenomic assembly may represent functionally similar gene sets that have diversified to elude anti-phage systems encoded by the host or other phage, perhaps helping to explain why bacteriophage populations show such extreme variation.

## Methods

### Iterative assembly pipeline

Here we first describe the basic steps of the optimized iterative de Bruijn graph assembly pipeline (available at https://sourceforge.net/projects/optitdba/), and then describe the implementation of each step in more detail. For each iteration, OPTITDBA 1) selects the optimal kmer, 2) generates a de Bruijn graph for the optimal kmer length, 3) removes the reads that map to the most highly abundant contigs from the dataset or reads that map to circular contigs, and 4) starts another iteration using all of the reads that do not map to those contigs. The loop ends when there are no highly abundant contigs meeting the criteria outlined below. At that point, all of the remaining reads will be assembled and mapped using the optimal values from the final iteration.

#### Selecting the optimal kmer

OPTITDBA assembles over a range of kmer values (63, 59, 55, 51, 47, 43, 39, 35, 31, 27, 23, and 19) using SOAPdenovo v1.05 [25] (flags: -p 10 -d 1 -M 3 -u -G 200 -R). All the steps taken to simplify the de Bruin graph, clipping tips, removing low-coverage links, resolving tiny repeats, and merging bubbles, were implemented as described in [25]. Each assembly is queried for whether any circular contigs longer than 2 kb were generated, suggesting complete assembly. If multiple kmer values resulted in circular genomes, than the largest such kmer value is selected.

If no such circles are generated, then the kmer values are scored by the length and depth of sequencing of its most abundant members. For each assembly, the contigs are sorted by the number of reads used to construct them and the cumulative length of the top 20 contigs is recorded. All contigs <1 kb in length are excluded. The kmer value with the longest cumulative length of its 20 most abundant is selected as the most optimal for that loop. The number of contigs selected (20) is arbitrary, can be specified by the user, and is used to balance computational resources against thorough assembly.

If more than 10^6^ reads are used as input, OPTITDBA randomly selects 10^6^ reads to use for the assembly trials. Preliminary tests indicated that this strategy reduced computation time at no detriment to optimal kmer determination.

#### Removing reads that map to the most highly abundant contigs

If no circular contigs are found, then the top 20 contigs from the assembly with the optimal kmer value are retained. If circular contig(s) is/are found, then the circular contig(s) and the top 20 contigs are retained. In pilot tests it was found that reducing the number of contigs that are retained at each step increases the final number of iterations as well as computational time, while the proportion of reads that were mapped was not impacted significantly.

The reads are then mapped to those retained contigs using BWA v0.5.8c [42]. The full set of reads is used to map, not the random subset described above (if used). All reads not mapping to these contigs are then used to start another iteration. The training set consisted entirely of paired reads, and both members of the pair were removed, even if only one mapped to a contig. We observed that when only one read in a pair mapped, the other often covered the junction of circular contigs, or gaps in the assembly. The cycle ends when either zero reads map, or there are zero contigs > = 1 kb in length.

While the iterative assembly pipeline developed here implements SOAPdenovo to perform assembly and BWA to perform mapping, the concept is independent of both programs.

### Benchmark sequences

The data used to benchmark this pipeline are those described in [3], [11]. Viral DNA was isolated from human fecal samples using sequential filtration and CsCl density ultra-centrifugation, then unprotected DNA was digested using DNaseI [43]. Viral DNA was subsequently recovered from particles, yielding a sample that was depleted in bacterial DNA by >100-fold (as measured by 16S rDNA qPCR [11]. Three samples each from six human subjects were extracted, pooled, and submitted for sequencing using the Illumina HiSeq 2000 platform (100 bp paired-end sequencing). Ten million reads were randomly selected from each dataset (except for Subject 6, which only had 5,754,268 reads) while preserving all read pairings, and assembled using either OPTITDBA, MetaIDBA v0.19 [29], or using SOAPdenovo with a kmer value of 63 and all of the same flags as in the iterative assembly pipeline. The kmer value of 63 for SOAPdenovo was found in previous tests to produce the highest N50 score using this dataset. MetaIDBA was run using default settings. See Table S1 for a summary of each dataset.

### Detection of Human Papillomavirus Virus

A previous analysis of these sequences found evidence of a single eukaryotic virus: Human Papilloma Virus type 6b [11]. Reads mapping to the HPV genome (NCBI gi: 9626053) were extracted and used to mix back in various quantities to a dataset that did not previously have any detected HPV sequences. The mixing was done by randomly selecting a total of 4*10^6^ reads for each test. The number of HPV reads varied across a range (500, 1000, 1500, or 1798 reads, corresponding to 6X, 13X, 19X, or 23X coverage), with three replicates of each. Each set of mixed reads was assembled using OPTITDBA, MetaIDBA, or SOAPdenovo as described above.

### Network analysis of viral proteins

In order to classify the assembled viral contigs according to their similarity with known proteins, we compared the predicted open reading frames (ORFs) on these contigs with 1) the RefSeq [44] collection of viral proteins, or 2) the Conserved Domain Database (CDD) [45] of conserved amino acid motifs. ORFs were predicted using Glimmer v3.02 [46], compared to Viral RefSeq (downloaded on 12/16/11) using blastp [47] (v2.2.25+, build 1/3/12), and compared to CDD [45] (downloaded on 10/18/11) using rpsblast (v2.2.25) [47]. Because of the difficulty of manually identifying patterns of similarity among contigs, we converted the protein similarity data into a format that could be viewed in the interactive network visualization tool Cytoscape [48]. In this bipartite network scheme, there are two classes of nodes: contigs and RefSeq proteins. When a RefSeq viral protein has a highly significant match (E<10^−50^) to an ORF encoded by a contig, a connection is made between those two nodes. For ease of visualization, we excluded all contigs that were either shorter than 3 kb, or had fewer than 5 hits to RefSeq proteins. The nodes and connections for all six datasets were combined and loaded into Cytoscape. The network was arranged using the spring-embedded layout (data available upon request).

### Protein family organization

In order to search for conserved protein families in a database-independent manner, we clustered the ORFs described above using UCLUST v1.2.22q [49]. Each of those protein families was compared to the Conserved Domain Database using rpsblast. Those protein families were next grouped into cassettes, meaning multiple protein families that can be found together on our contigs. Cassette discovery proceeded in the following manner. Each protein family was classified according to the list of contigs that encoded it. Next, all of the protein families were compared, seeing how many of those occurred on common contigs. A given pair of protein coding families was grouped into a cassette when the smaller of the two families was found on a shared contig at least 80% of the time. This process was performed iteratively, recalculating the overlap scores after each pair of protein families was merged together. In subsequent iterations, protein families could also merge in the same way with cassettes that formed earlier.

If a pair of proteins formed a cassette found on multiple contigs, we expect shared ORFs to be in the same relative orientations. To calculate the consistency of orientation across contigs, we used a simple co-orientation score, calculated in the following way. Any two genes have four possible relative orientations. For every pair of protein clusters in a module, we calculate the proportion of contigs that contain the orientation found most commonly. Discovery and analysis of protein modules was implemented in an R script that is available along with the iterative assembly pipeline, at https://sourceforge.net/projects/optitdba/.

### Computation

Computation was carried out on a home-built computer with 192 Gb of RAM and 12 cores (24 hyperthreaded). The computer was assembled from parts costing $16,060 (USD) (a full parts list is available at http://microb230.med.upenn.edu/protocols/comput_resources.html). Computation times for assembly of single viral communities (5.7*10^6^–10^7^ reads) using this pipeline were 20.7 to 132.1 wall clock hours, with a median of 39.0 hours. The computation time may vary with the community complexity and number of reads, as the dataset with the longest compute time (#1: 132.1 hours), also had the largest number of predicted species by PHACCS (data not shown), the dataset with the shortest compute time (#6: 20.7 hours) was the only one to have less than 10^7^ sequences, and all of the other datasets ranged between 38 and 56 hours.

## Supporting Information

Figure S1 **Optimized iterative de Bruijn graph assembly of 10^7^ viral metagenomic sequences.** A) Summary of run statistics for each iteration of the assembly, in which reads mapping to newly assembled contigs were removed at each iteration. The horizontal axis indicates the iteration number. For each of those iterations, the vertical axes indicate the number of reads remaining at the end of the iteration, the number of reads mapped during that iteration, the number of contigs made, the number of circular contigs made, and the optimal kmer chosen for that iteration. B) Characteristics of contigs by iteration of assembly. Each point is a contig with a length shown on the horizontal axis, depth of the assembly is shown on the vertical axis, and the iteration at which it was assembled indicated by color. The contigs that were assembled at earlier cycles (shown with bluer points) are generally longer and more deeply sequenced.
(PDF)

Figure S2 **Comparison of assembly methods by known genome reconstruction.** Shotgun sequences from HPV Type 6b were extracted from one dataset and added back to another dataset lacking HPV in varying amounts, as indicated in the grey boxes above each plot. The success of HPV reconstruction was measured as the length of the longest HPV-matching contig as a proportion of the total HPV length (vertical axis). The horizontal axis indicates the three assembly methods used. Three independent random samples were created for each level of coverage, and the assemblies using the same dataset are connected with a line.
(PDF)

Figure S3 **One additional example of phage cassette.** Contigs are shown as horizontal black lines, ORFs on those contigs are shown by black arrows above and below those lines, and the organization of those ORFs into protein-coding families is shown with colored boxes. The subject that each contig was assembled from is shown on the left of each panel. When a protein-coding family was functionally annotated according to its similarity with the CDD, that annotation is listed in the legend.
(PDF)

Table S1 **Assembly statistics.**
(DOC)
