# Supplementary figures and images for: Conservation of Gene Cassettes among Diverse Viruses of the Human Gut
Source: PLoS One. 2012 Aug 10;7(8):e42342. doi: 10.1371/journal.pone.0042342 (PMC3416800; doi:10.1371/journal.pone.0042342)

**Fig S1**

**A**

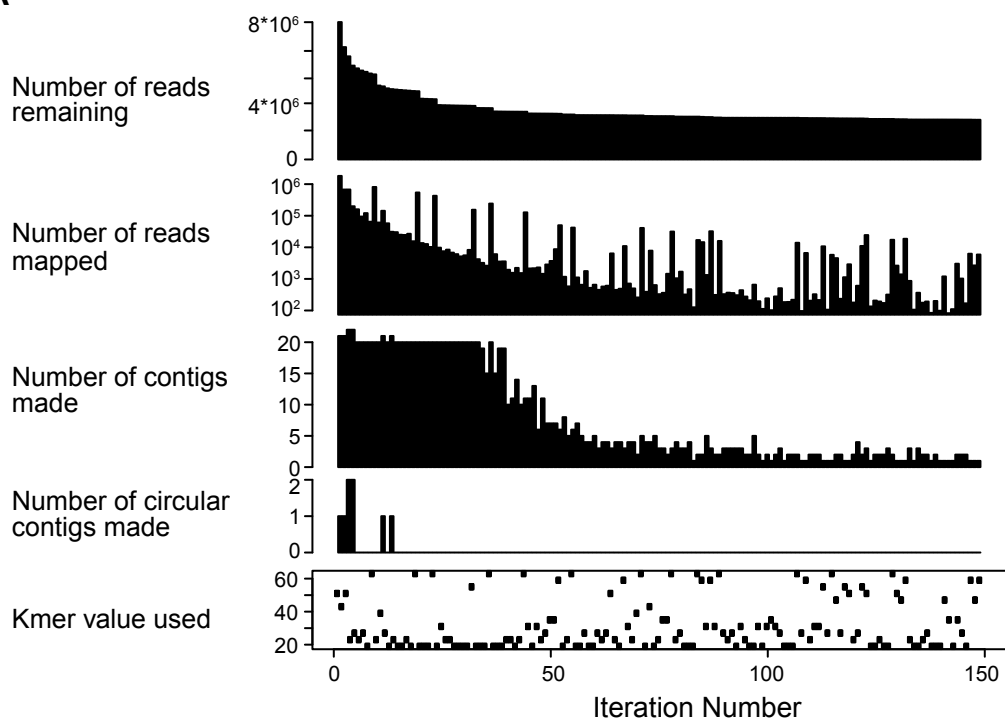

**B**

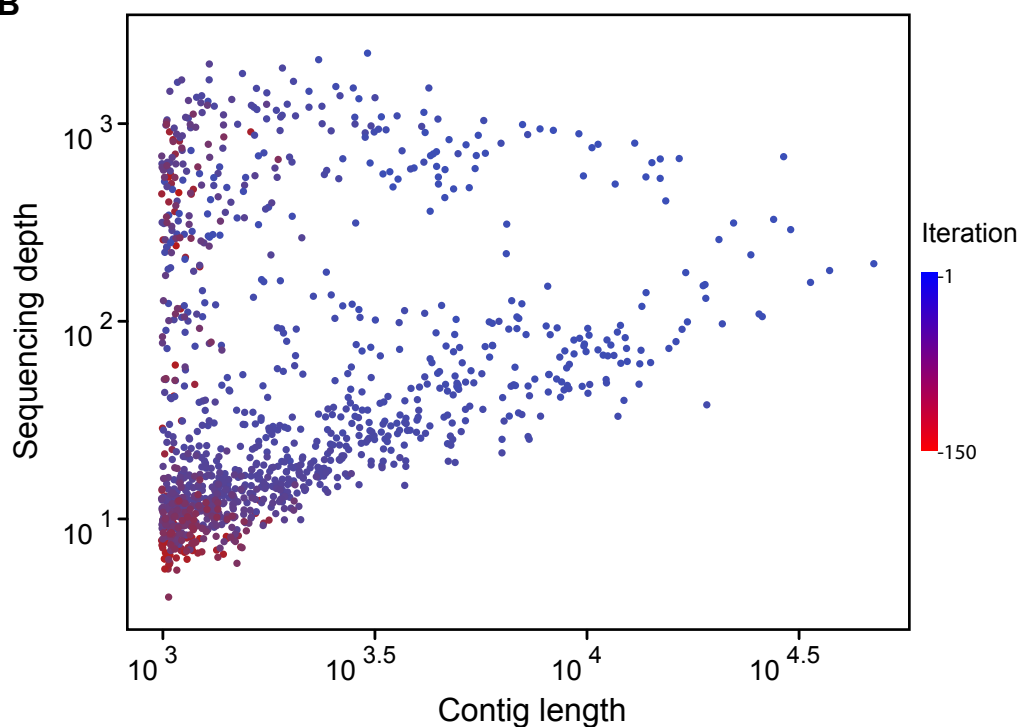

Supplement: Figure S1 — Optimized iterative de Bruijn graph assembly of 107 viral metagenomic sequences. A) Summary of run statistics for each iteration of the assembly, in which reads mapping to newly assembled contigs were removed at each iteration. The horizontal axis indicates the iteration number. For each of those iterations, the vertical axes indicate the number of reads remaining at the end of the iteration, the number of reads mapped during that iteration, the number of contigs made, the number of circular contigs made, and the optimal kmer chosen for that iteration. B) Characteristics of contigs by iteration of assembly. Each point is a contig with a length shown on the horizontal axis, depth of the assembly is shown on the vertical axis, and the iteration at which it was assembled indicated by color. The contigs that were assembled at earlier cycles (shown with bluer points) are generally longer and more deeply sequenced. (PDF) [file pone.0042342.s001.pdf]

**Fig S2**

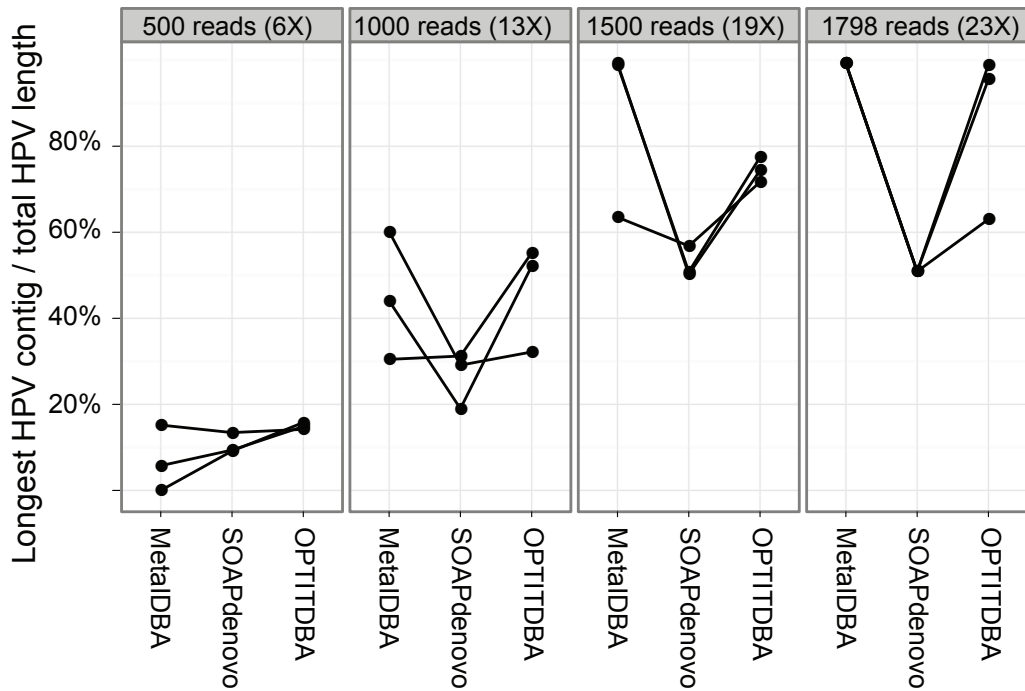

Supplement: Figure S2 — Comparison of assembly methods by known genome reconstruction. Shotgun sequences from HPV Type 6b were extracted from one dataset and added back to another dataset lacking HPV in varying amounts, as indicated in the grey boxes above each plot. The success of HPV reconstruction was measured as the length of the longest HPV-matching contig as a proportion of the total HPV length (vertical axis). The horizontal axis indicates the three assembly methods used. Three independent random samples were created for each level of coverage, and the assemblies using the same dataset are connected with a line. (PDF) [file pone.0042342.s002.pdf]

**Fig S3**

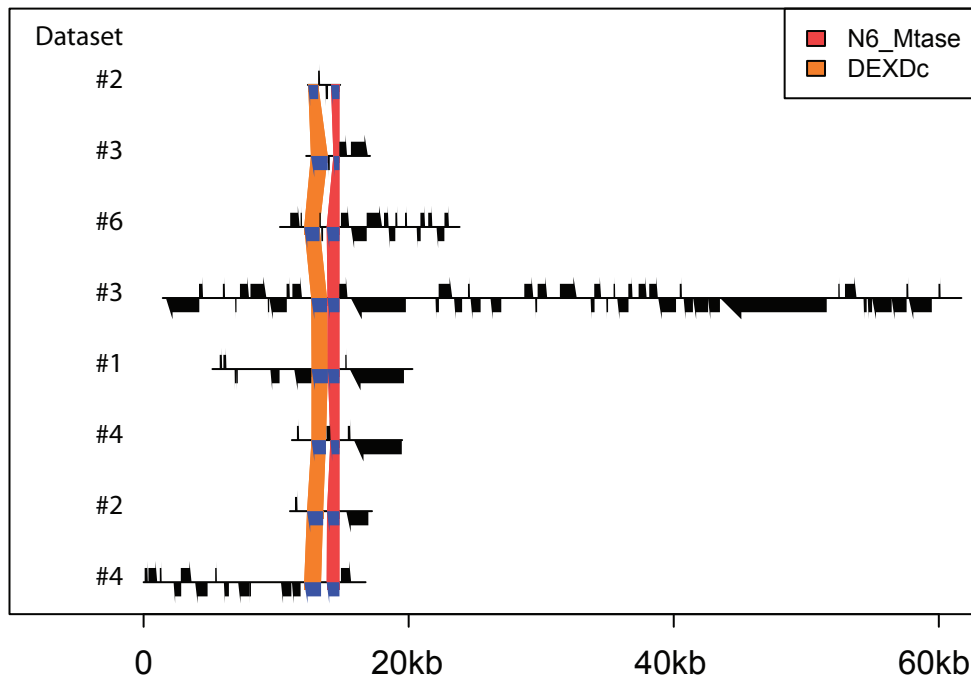

Supplement: Figure S3 — One additional example of phage cassette. Contigs are shown as horizontal black lines, ORFs on those contigs are shown by black arrows above and below those lines, and the organization of those ORFs into protein-coding families is shown with colored boxes. The subject that each contig was assembled from is shown on the left of each panel. When a protein-coding family was functionally annotated according to its similarity with the CDD, that annotation is listed in the legend. (PDF) [file pone.0042342.s003.pdf]
