# Supplementary material for: Conservation of Gene Cassettes among Diverse Viruses of the Human Gut
Source: PLoS One. 2012 Aug 10;7(8):e42342. doi: 10.1371/journal.pone.0042342 (PMC3416800; doi:10.1371/journal.pone.0042342)
Supplement: Table S1 — Assembly statistics. (DOC) [file pone.0042342.s004.doc]

**Supporting Table 1. Assembly statistics.**

|  |  | **Optimized iterative assembly pipeline (OPTITDBA)** | | | | **SOAPdenovo** | | | |  |
| --- | --- | --- | --- | --- | --- | --- | --- | --- | --- | --- |
| Subject | Number of reads | Number of contigs | Longest Contig (bp) | N50 (bp) | Circular | Number of contigs | Longest Contig (bp) | N50 (bp) | Circular | Reference |
| 1 | 10000000 | 3516 | 58746 | 2981 | 22 | 1565 | 108497 | 5649 | 3 |  |
| 2 | 10000000 | 1400 | 42939 | 3415 | 10 | 960 | 93814 | 7605 | 2 |  |
| 3 | 10000000 | 977 | 60257 | 3986 | 8 | 487 | 45986 | 5289 | 1 |  |
| 4 | 10000000 | 1617 | 44975 | 3661 | 16 | 805 | 40190 | 6165 | 2 |  |
| 5 | 10000000 | 1150 | 47449 | 4561 | 16 | 463 | 117255 | 13073 | 2 |  |
| 6 | 5754268 | 588 | 77186 | 5772 | 10 | 340 | 47138 | 9053 | 2 |  |

Note: All contigs shorted than 1,000bp are omitted from the above table.

Reference

1. Minot S, Grunberg S, Wu GD, Lewis JD, Bushman FD (2012) Hypervariable loci in the human gut virome. Proc Natl Acad Sci U S A.
